# Talks with Dr. Mandeville

**Published:** 1892-02

**Authors:** 


### TALKS WITH DR. MANDEVILLE.

### No. 1.

It is claimed by many physicians, Doctor, that a vegetable diet is best suited to persons of a nervous temperament; what is your opinion in regard to it?

It is all nonsense. Why, the entire human anatomy would have to undergo a change to make man an herbivorous animal. The digestive apparatus of the vegetable feeders is far more complex in arrangement than that of carnivorous creatures, or of human beings. The stomach of a cow, for instance, is arranged in four parts—four stomachs, in fact—through each of which the food must pass before returning to the mouth to be still further masticated, before it can be digested. Flesh eating animals have but one stomach like a hollow sack. One killed three or four hours after it has eaten a full meal will be found to have an empty stomach, while in the case of an ox or sheep, at some length of time after eating, the process of digestion will be found to have hardly finished its first stage. It is apparent from this, if from no other evidence, that human stomachs, especially of weak or nervous persons, should be saved the labor of converting vegetable substances into a form fit for assimilation so long as they can be supplied with animal food.

I am disposed to exclude vegetables, with the exception of cereals and a little fruit, entirely from the diet of nervous persons. First, because animal food is more nutritious, not only to the nervous system but to the body generally. It has all the essential elements for the formation of the tissues of the body and is easily digested. It is the natural sustenance for human beings, for it is possible for one to subsist upon it alone, in any climate, and continue in a normal condition. The first food taken by the infant man—milk—is strictly an animal substance. It contains all the elements necessary to the growth of the human body and to its maintenance in a state of health. This cannot be said of any one vegetable food. Then the nervous system consists largely of fat, and this substance must be supplied in some manner in order that the brain and other nerve structures shall be properly nourished. If a person uses up his brain faster than he makes it, he soon becomes irritable and nervous, and if he does not assimilate enough food to supply its demands, his mind is sure to become weak. The healthiest and strongest individuals even, should eat a far greater portion of meat than of vegetable food. Beef should be taken as the standard meat. It answers every purpose of the system when not cooked too much. Veal and pork are not so easily digested, but pork, so far as composition goes, is an excellent food for nervous persons. As a rule, salt meat is not adapted to their requirements. The nutritious juices, to a great extent, go to the brain, and the flesh of young animals digests more readily than that of mature ones. This is also true of the flesh of wild birds, which is more tender than that of domesticated ones, owing to the greater amount of exercise they take, thereby renewing their flesh more rapidly and making it younger than that of birds whose habits are less active, a hint that might be of benefit to ladies of sedentary habits, who are desirous of prolonging an appearance of youth. Fish of all kinds are good for nervous people. Raw eggs, contrary to the general opinion, are not so digestible as those which have been cooked. They should be boiled just enough to moderately harden their whites. Fried eggs, or as to that matter, any thing else fried, should not be eaten by nervous people.

The first thing for one who has become exhausted by mental work, or who has been born weak and irritable, is to furnish his brain with sufficient food to repair the damage it has sustained and build it up into a strong, healthy condition. Such persons usually suffer from nervous dyspepsia. Their stomachs are inadequate to the task of digestion. Owing to a deficient nerve power food lies there unacted upon by the gastric juice, because there either is none, or an insufficient supply to do its office ; hence food instead of helping to renew the body and strengthen the nervous system, undergoes fermentation and the body it should nourish, starves, leaving one in a worse state than if the food had not been eaten, for the fermentation develops acids and gases, detrimental to good health. Whilst nervous people may get all the fat they need out of sugar and starch, it is better for those whose digestive organs are very weak, or whose nerves are in a highly sensitive condition to get it from the animal kingdom, than to compel their enfeebled stomachs, intestines, and pancreas, to create it out of less assimilable substances. Good bread with plenty of sweet butter is an excellent food for the nerves.

Persons troubled with insomnia, nervous starting from sleep, and sensations of falling, can often be cured by limiting themselves, for a time, to a diet of milk ; I mean milk and nothing else. An adult requires about three pints or two quarts daily. Moderate draughts of water aid the digestion of food by making it soluble, besides having a tonic effect, but hot water should never be taken into the stomach, for nothing is more relaxing to it.

With proper eating and drinking you would have fewer broken down nervous wrecks and far more vigorous intellects, but the human species cannot eliminate flesh from its food and maintain a healthy organism The notion that nothing but vegetables should be eaten is one that is apt to overtake persons in life, but it is due to some disorganization and is usually of short duration. The fancy is more apt to assail the young than the middle aged, and females more frequently than males; but meat is the food of humanity, and man must stick to it.

